# Impact on Fecal Microbiota and Health-Related Markers of an Intervention Focused on Improving Eating Behavior in People at Risk of Food Insecurity

**DOI:** 10.3390/nu15163537

**Published:** 2023-08-11

**Authors:** Aida Zapico, Silvia Arboleya, Nuria Salazar, Carmen Perillán, Sergio Ruiz-Saavedra, Clara G. de los Reyes-Gavilán, Miguel Gueimonde, Sonia González

**Affiliations:** 1 Department of Functional Biology, University of Oviedo, 33006 Oviedo, Spain; aida.zapico@ispasturias.es (A.Z.); perillanmaria@uniovi.es (C.P.); 2 Diet, Microbiota and Health Group, Instituto de Investigación Sanitaria del Principado de Asturias (ISPA), 33011 Oviedo, Spain; silvia.arboleya@ipla.csic.es (S.A.); nuriasg@ipla.csic.es (N.S.); sergio.ruiz@ipla.csic.es (S.R.-S.); greyes_gavilan@ipla.csic.es (C.G.d.l.R.-G.); mgueimonde@ipla.csic.es (M.G.); 3 Department of Microbiology and Biochemistry of Dairy Products, Instituto de Productos Lácteos de Asturias (IPLA-CSIC), 33300 Villaviciosa, Spain

**Keywords:** vulnerable subjects, sensorial perception, gut microbiota, processed food, depression, sustainable diet

## Abstract

Non-communicable diseases are particularly prevalent among low-income individuals and are associated with the consumption of processed foods, fat, and sugars. This work aims to evaluate the impacts of a nutrition education intervention for low socio-economic individuals on sensory perception, health-related parameters and gut microbiota. Twenty low-income adults underwent a 4-week intervention. Dietary information (three 24 h recalls), detection thresholds and discrimination scores (salty and sweet), and severity of depressive symptoms (Beck Depression Inventory-II (BDI-II)) were collected. Fecal microbial composition and short chain fatty acids were determined by 16S ribosomal RNA-gene sequencing and gas chromatography, respectively. After the intervention, 35% of subjects presented higher compliance with dietary recommendations, increased consumption of vegetables and lignans and reduced consumption of processed meats and nitrosamines, together with depleted levels of Actinomycetota. Higher discrimination for salty and sweet and lower BDI-II scores were also obtained. This nutrition education intervention entailed changes in dietary intake towards healthier food options, reduced potentially carcinogenic compounds and improved scores for discrimination and severity of depressive symptoms. The confirmation of these results in future studies would enable the design of strategic policies contributing to the optimal nutrition of materially deprived families through affordable healthy plant-based interventions.

## 1. Introduction

Solid evidence demonstrates the importance of a healthy lifelong dietary pattern for maintaining overall health status. The growing prevalence of non-communicable diseases worldwide has been associated with the loss of traditional eating patterns and the increased consumption of ultra-processed foods, fat, salt, and refined sugar in the diet [1]. While this situation is widespread among the population in developed countries, it is particularly striking in some groups, such as those at risk of vulnerability who receive food aid [2]. Socio-economically deprived groups exhibit a suboptimal eating behavior, associated with the higher cost of healthy foods and inadequate knowledge and skills in relation to healthy food choice and food preparation [3,4,5,6,7,8]. In addition, poor lifestyle habits, such as sedentary behavior or smoking, and stress have frequently been reported in these subjects [9,10,11,12,13]. 

Recent literature has shown that processed foods have a negative impact on dietary quality and health due to the poor nutrient density, their high additive content, and association with higher intakes of sugars and trans-fatty acids [14]. Sugar-sweetened beverages and processed meats [14,15] have been associated with an increased risk of non-communicable diseases including colorectal cancer [16,17,18]. Along with that, the scarce consumption of fruits, vegetables and whole grains may lead to a reduced intake of fiber or (poly)phenols, which have been identified as key players in microbiota modulation in addition to their anti-inflammatory and antioxidant properties [17,19,20].

Over the last decades, a consensus has been reached on the impact of microbiota alteration in host health [21]. The transition from a Westernized diet to a Mediterranean-type pattern, for 4 weeks, with constant energy intake and physical activity has shown noticeable changes in the intestinal microbiome in people with cardiometabolic risk, with these changes being proportional to the degree of adherence [22]. Regarding individual dietary components, a higher intake of whole grain cereals was associated with an increase in Bifidobacteria in healthy humans [23], whereas a high salt ingestion in experimental animals was linked to a decrease in *Lactobacillus* and Prevotellaceae and to an enrichment in Erysipelotrichaceae and Oscillospiraceae [24,25]. While Erysipelotrichaceae has been associated with inflammation [26], higher levels of bifidobacteria and lactobacilli are recognized as beneficial for human health, improving outcomes such as obesity and depression [27,28,29]. Some authors have shown an increase in the microbial diversity index with Mediterranean diet (MD) interventions, although others have found the opposite or no association [21]. Throughout the gut–brain axis, microbial metabolites, and immune, neuronal, and metabolic pathways could drive dietary modulation [30]. It is possible that the high prevalence rates of stress, anxiety, and depression, described in people at risk of socio-economic vulnerability, may be associated with unhealthy food preferences. Foods with a high content in sugars and fats have been associated with a self-rewarding effect in response to the increase in cortisol levels [31,32,33]. In this regard, dietary interventions with a high fiber diet in obese females had a positive impact on stress [34] and depression [35] linked to changes in the abundance of some beneficial species from the intestinal microbiota such as *Bifidobacterium longum* [36].

Based on this evidence, the objective of this study was to evaluate the impact of an educational and dietary intervention on sensorial perception, health-related parameters and the composition and activity of the intestinal microbiota in a group of subjects in a socio-economically vulnerable situation. 

## 2. Materials and Methods

### 2.1. Participants and Recruitment

The MESAS (Economic, Healthy, and Sustainable Menus) project consists of an educational and dietary program promoted by the Alimerka Foundation for low-income individuals belonging to local assemblies of the Red Cross of Asturias (northern Spain). Volunteers were recruited from the Red Cross of Asturias and informed about the objectives of the study. An informed written consent was obtained before enrolment. Exclusion criteria were to be diagnosed with any gastrointestinal chronic condition or to have consumed antibiotics in the last month.

The Ethical Committee of the Hospital Universitario Central de Asturias (CEImPA2021.307) approved the whole procedure and methodology of this project. The procedures were performed in accordance with the fundamental principles set out in the Declaration of Helsinki, the Oviedo Bioethics Convention, and the Council of Europe Convention on Human Rights and Biomedicine, as well as in Spanish legislation on Bioethics. Directive 95/46/EC of the European Parliament and the Council of October 1995 on the protection of individuals regarding the processing of personal data was strictly followed. 

### 2.2. Study Design

**Figure 1:**
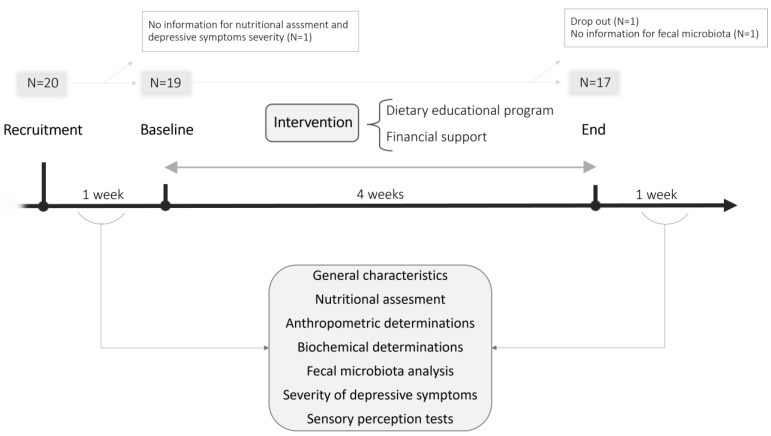
Scheme of study design and timeline.

Twenty volunteers were recruited and scheduled for a baseline face-to-face interview conducted by trained interviewers and for blood collection in the week before the dietary and educational intervention. For the collection of fecal samples, participants were provided with sterile containers and detailed instructions (Figure 1). For the initial sample size and considering microbial relative abundances, the statistical power of our results with a type I error probability of 0.05 was 95–98% (Power and Sample Size Calculation version 3.0.43 (Vanderlbilt University, Nashville, TN, USA)).

The intervention consisted of a 1 h educational intervention explaining the basis of a healthy and affordable diet based on the consumption of locally produced fresh products. This activity was complemented with financial support for the acquisition of fresh products provided by Alimerka Foundation, along with dietary education materials (general dietary recommendations, a monthly meal plan, recipes and a shopping list adjusted to the budget). This allowed them to complement the assistance that they receive regularly with non-perishable foodstuffs through the Asturias Red Cross. The material used in the intervention was developed specifically for the purpose of this project and is available at: https://www.fundacionalimerka.es/wp-content/uploads/2022/01/MESAS_guia-completa.pdf (accessed on 18 May 2022) in Spanish language.

At baseline and at the end of the study, general characteristics of the sample population, nutritional assessment, anthropometric determinations, blood analyses and fecal sample collection were assessed, together with a self-completed depression test and sensory perception assays. Participants were encouraged to contact the interviewer if they had any questions or concerns during the study. Seventeen subjects completed the intervention.

### 2.3. General Characteristics

Information about age, gender, educational level, family size, as well as questions related to energy expenditure, smoking habit, alcohol consumption and the presence of chronic conditions were included in the questionnaire.

The highest educational level attained by each volunteer was registered and classified into primary, secondary, technical or university higher education. The physical activity of the previous week was quantified by using the International Physical Activity Questionnaire (IPAQ) [37,38]. The total metabolic equivalent of task (MET) and the IPAQ classification into three categories of physical exercise were obtained [38]. 

### 2.4. Nutritional Assessment

Information regarding the dietary intake of the participants was collected during the face-to-face interviews of no more than 30 min of duration through three nonconsecutive 24 h recalls. In addition, information on cooking habits (boiled, fried, grilled, baked/broiled, or barbecued) and the part of the food that was finally consumed (breast or thigh in the case of chicken) as well as the possible consumption and/or cooking of the skin (cooking with skin and eating the skin; cooking with skin, but not consuming it; and cooking without skin) were included. Standardization of the information reported was achieved by using photographs of different serving sizes and others in which the degree of browning increased progressively, as has been previously reported [18,39].

Food consumption was classified into food groups according to the Centre for Higher Education in Nutrition and Dietetics (CESNID) criteria. CESNID food composition tables were used to transform food consumption into energy and macronutrients intake [40]. United States Department of Agriculture (USDA) and Marlett and Cheung tables were used to calculate the starch and fiber content, respectively [41,42]. (Poly)phenols were extracted from Phenol Explorer version 3.6. [43]. Regarding the consumption of food-derived xenobiotics, the European Prospective Investigation into Cancer and Nutrition (EPIC) Potential Carcinogen Database was the main data source for the content of heterocyclic amines, polycyclic aromatic hydrocarbons (PAHs), nitrates, and nitrites per g of food [44]. When necessary, missing information was completed by additional sources such as the Computerized Heterocyclic Amines Resource for Research in Epidemiology of Disease (CHARRED) database [45], the European Food Safety Authority (EFSA) data [46], the U.S. Food and Drug Administration (FDA) composition tables [47] and others [48,49,50,51,52,53,54,55,56].

The validated 14-point MD screener “PREvención con Dieta MEDiterránea” (PREDIMED) was used to assess adherence to a Mediterranean pattern [57,58]. The degree of compliance with the dietary intervention was evaluated based on the number of PREDIMED criteria that improved with the intervention. Those subjects that improved 2 or fewer criteria were classified as lower compliance (LC) while those who improved 3 or more items were considered as higher compliance (HC).

### 2.5. Severity of Depressive Symptoms

Depressive symptoms severity was estimated using the validated 21-item Spanish Beck Depression Inventory-II (BDI-II) [59]. Volunteers rated each item on an intensity scale ranging from 0 to 3, with a maximum possible score of 63 points. The severity of depressive symptoms was categorized as minimal (0–13 points), mild (14–19), moderate (20–28), or severe (29–63) using previously established references [59,60].

### 2.6. Anthropometric Determinations

Height (m) and weight (kg) were assessed by standardized protocols [61] and body mass index (BMI) was calculated using the formula: weight/(height)^2^. Spanish Society for the Study of Obesity (SEEDO) criteria [62] were used to classify subjects as normal weight (18.5–24.9 kg/m^2^), overweight (25.0–29.9 kg/m^2^), and obese (≥30.0 kg/m^2^). For body fat percentage, bioelectrical impedance in a calibrated TANITA device (Tanita Corporation of America, Inc., Arlington Heights, IL, USA) was employed, and waist and hip circumferences were measured using an inelastic and extensible tape, as indicated by standard criteria [63]. 

### 2.7. Biochemical and Microbiological Analysis 

Twelve-hour fasting blood samples were drawn by venipuncture and collected in separate tubes for serum and plasma. The samples were kept on ice and centrifuged (1000× *g*, 15 min) within 2–4 h after collection. Plasma and serum aliquots were stored at −20 °C until analyses. From the blood samples, the biochemical parameters fasting plasma glucose, cholesterol, high- and low-density lipoproteins (HDL and LDL), triglycerides, uric acid, creatinine and iron were determined by standard methods in external laboratories. 

Fecal samples were collected within ±24 h of blood collection in sterile containers supplied to each volunteer along with the instructions for sample collection. The samples were frozen after deposition within a period not exceeding two hours and transported to the laboratory. Fecal sample specimens were diluted 1/10 (*w*/*v*) in sterile PBS solution and homogenized at full speed in a LabBlender 400 stomacher (Seward Medical, London, UK) for 3 min. The samples were centrifuged for 15 min at 4 °C and 17,530× *g* and the obtained supernatants were separated from the pellets and kept frozen at −20 °C until use. From the pellet obtained, DNA was extracted in accordance with the Q Protocol for DNA extraction defined by the International Human Microbiome Standards Consortium [64] using the QIAamp Fast DNA Stool Mini Kit (Qiagen, Sussex, UK). The quantification of extracted/purified DNA and the 260/280 ratio were performed using the Take3 Micro-Volume plate and Gen5 microplate reader (BioTek Instrument Inc., Winooski, VT, USA). The DNA obtained was kept frozen at −80 °C until analysis. 

The variable region V3–V4 of the bacterial 16S rRNA genes present in each fecal community was amplified by PCR and the resulting amplicons were sequenced on an Illumina NovaSeq 6000 platform instrument (San Diego, CA, USA). The obtained individual sequence reads were filtered to remove low quality sequences. All Illumina quality-approved, trimmed, and filtered data were integrated to generate de novo 16S rRNA Operational Taxonomic Units (OTUs) with ≥97% sequence homology using Uparse software (Uparse v7.0.1090). A classification of all reads to the lowest possible taxonomic rank was performed using Quantitative Insights Into Microbial Ecology (QIIME) and a reference dataset from the SILVA 138 database. The whole procedure of sequencing and annotation was performed at Novogene Bioinformatics Technology Co., Ltd., Cambridge, UK. 

Short chain fatty acids (SCFA) were analyzed by gas chromatography from the supernatants of 1 mL of the homogenized feces [65]. A chromatograph 6890N (Agilent Technologies Inc., Palo Alto, CA, USA) connected to a mass spectrometry detector (MS) 5973N (Agilent Technologies) and a flame ionization detector (FID) was used for SCFA identification and quantification, as described in previous works [66].

### 2.8. Salt and Sweet Sensitivity and Discrimination

Our survey was conducted in early summer in Asturias (Spain) with an average room temperature of 20 °C. The sensitivity and discrimination tests were specifically designed for this research, using concentrations already validated in the literature [67,68,69]. 

For the sensory perception tests, NaCl and sucrose were dissolved in mineral water (low mineral content) to create salty and sweet tastes, respectively. Throughout the assessment, plain water was offered for mouth rinsing and the order in which the solutions were tasted was freely chosen. Taste solutions were kept in the dark at 5 °C when not in use. 

A test based on exposure to 5 different concentrations of sucrose was designed, including the detection and recognition thresholds developed by Webb et al., (0, 5, 15, 30 and 21,950 mM) [69]. Each concentration was anonymized with a random number, with volunteers having to report detection or not of sweet taste for the sensitivity tests and ordering the 5 concentrations from lowest to highest in order to determine the discrimination of each taste. For each correct answer, 1 score was given. The same procedure was carried out with NaCl for salty taste with 5 different concentrations, including the detection and recognition thresholds developed by Malaga et al., (0, 5, 10, 15 and 50 mM) [68].

Sensory perception tests at baseline and after the intervention were completed by 80% of the individuals selected for this study (n = 14).

### 2.9. Statistical Analysis

IBM SPSS software version 25.0 (IBM SPSS, Inc., Chicago, IL, USA) was used to analyze all the collected data. Goodness of fit to the normal distribution was checked by means of the Kolmogorov–Smirnov test, and as normality of the variables was not achieved, non-parametric tests were used. Categorical variables were presented as number and percentage (n (%)) and continuous variables as the median and 25th and 75th percentiles (P_25_–P_75_) or mean ± SD. For categorical variables Mc Nemar and Fisher tests were performed, and continuous variables were analyzed using the Wilcoxon and Mann–Whitney U tests with Bonferroni correction, for comparisons within each group (T0 vs. T1) and between groups (T0 vs. T0 and T1 vs. T1), respectively (*p* value < 0.05). GraphPad Prism 8 (La Jolla, CA, USA) was used for graphical representations.

## 3. Results

### 3.1. Characteristics of the Study Sample

The general baseline characteristics of the sample are shown in Table 1. The sample comprised 82% adult women (median age 40 years old), and 35% and 24% of the study sample presented occasional alcohol consumption and smoking habit, respectively. 

### 3.2. Effect of the Intervention on Dietary and Nutritional Intake

**Figure 2:**
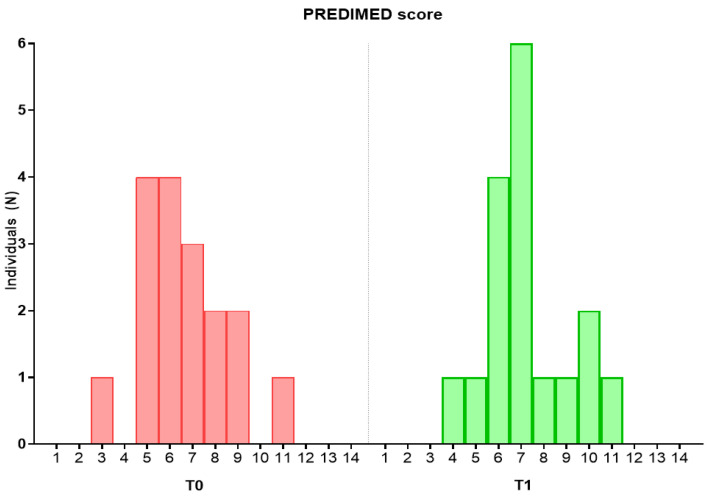
“PREvención con DIeta MEDiterránea” (PREDIMED) score of the sample across the study. T0, baseline; T1, end.

Differences in the adherence to MD during the study are shown in Figure 2. Higher scores in PREDIMED were observed with intervention. The impact of the intervention on the daily intake of energy and major food groups for both LC and HC groups is shown in Table 2. The HC group significantly increased the daily intake of vegetables (144 to 251 g/day) and decreased the consumption of cereal products (118 to 57 g/day) and processed meats (12 to 0 g/day). Regarding bioactive compounds, subjects with HC showed lower consumption of starch (35 to 2 g/day) and higher consumption of lignans (10 to 32 g/day) after the intervention (Table 3). In addition, the intervention resulted in a reduced level of food-derived xenobiotic compounds in the HC group (Table 4), specifically dibenzo (a) anthracene (4 to 2 ng/day), acrylamide (8 to 3 µg/day), total PAHs (1108 to 373 ng/day) and n-nitrosopiperidine (28 to 0 ng/day). On the other hand, the consumption of nitrates was increased in both groups after the intervention (30 to 67 mg/day and 101 to 153 mg/day, in the LC and HC, respectively).

### 3.3. Effect of the Intervention on Anthropometric and Biochemical Parameters, Severity of Depressive Symptoms and Sensitivity and Discrimination for Salt and Sweet Taste

**Figure 3:**
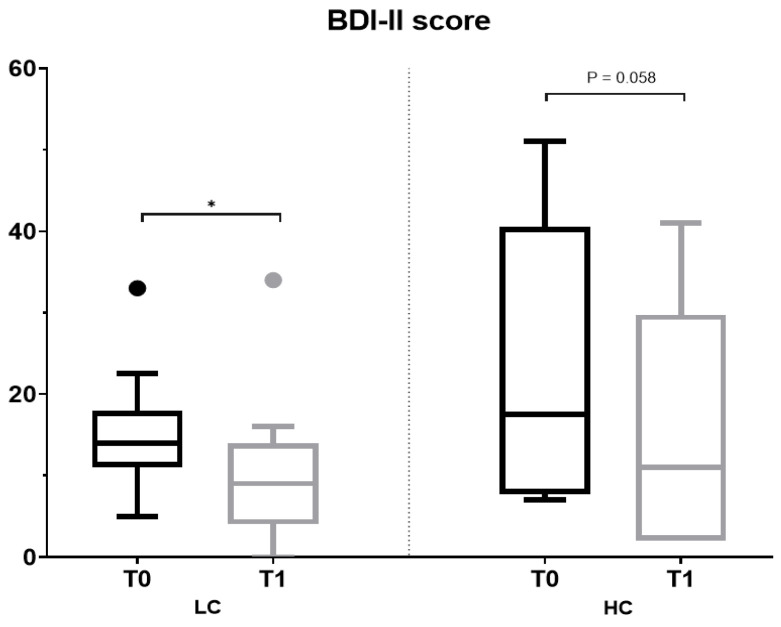
Analysis of Beck Depression Inventory-II (BDI-II) total score at baseline and after the intervention by the degree of compliance with the dietary intervention. (*) Statistical differences were found by Wilcoxon test for comparisons within each group (T0 vs. T1) (*p* value < 0.05). HC, higher compliance; LC, lower compliance; T0, baseline; T1, end.

**Figure 4:**
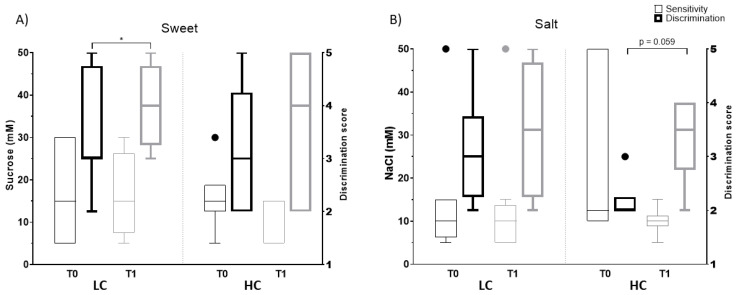
Analysis of sensitivity thresholds (left Y axis) and discrimination scores (right Y axis) for sweet (**A**) and salt (**B**) at baseline and after the intervention by the degree of compliance with the dietary intervention. Statistical differences were found by Wilcoxon (*) for comparisons within each group (T0 vs. T1) (*) (*p* value < 0.05). HC, higher compliance; LC, lower compliance; T0, baseline; T1, end.

No significant changes were found in anthropometric values (Table S1) or biochemical parameters (Table S2) after the intervention except for total body fat, which increased in volunteers with HC (32 to 35%). The severity of depressive symptoms was improved in both the LC and HC groups. The proportion of individuals with an ameliorated BDI-II total score for depressive symptoms after the intervention was higher in the HC group (83 vs. 64%), whereas a significant lower BDI-II score after the intervention was only found in the LC group (score change from 14 to 9) (Figure 3, Table S3). Regarding the sensory tests, no significant differences were observed in salt and sweet detection thresholds after the intervention, whereas discrimination scores were increased in both LC and HC groups (Figure 4, Table S4). The proportion of individuals with a higher discrimination score after the intervention was higher in the HC group (67 vs. 50%), although the observed changes reached statistical significance only for sweet taste in the LC (score change from 3 to 4) (Figure 4, Table S4).

### 3.4. Effect of the Intervention on the Fecal Profile of Microbiota and SCFA

**Figure 5:**
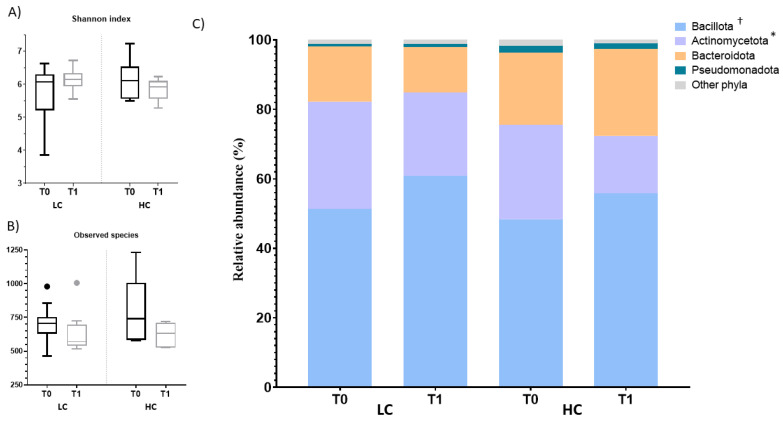

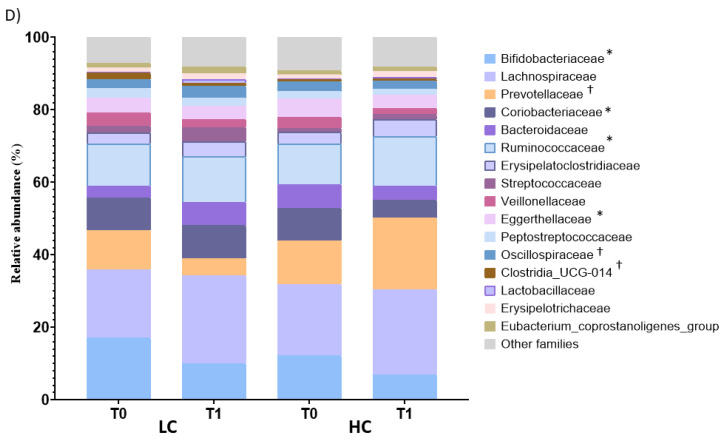
Analysis of microbiota diversity indexes and microbiota relative abundance profile composition at baseline and after the intervention by the degree of compliance with the dietary intervention. Box plot of (**A**) Shannon and (**B**) Observed species. No significant differences were found by Wilcoxon test (*p* value < 0.05). Microbiota relative abundance distribution to (**C**) phylum and (**D**) family level. Statistical differences were found by Wilcoxon for comparisons within each group (T0 vs. T1) in the LC (†) and HC (*) (*p* value < 0.05). Only taxa with relative abundance greater than 1% in mean values and in at least two samples are presented. HC, higher compliance; LC, lower compliance; T0, baseline; T1, end.

**Figure 6:**
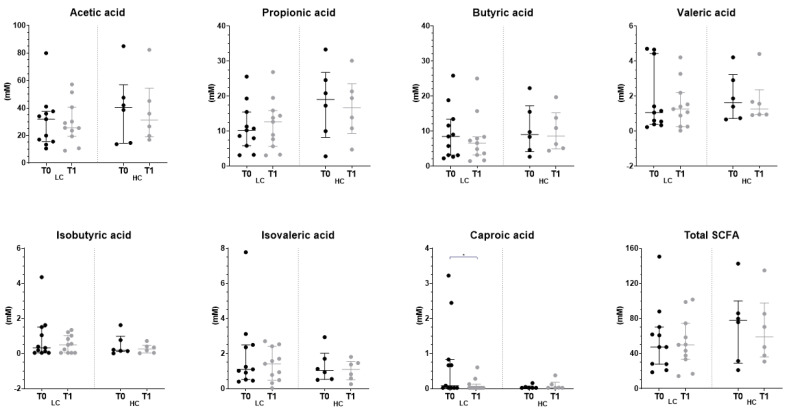
Analysis of levels of fecal SCFA at baseline and after the intervention according to the degree of compliance with the dietary intervention. Box plot data are represented as median and interquartile range. Statistical differences were found by Wilcoxon (*) for comparisons within each group (T0 vs. T1) (*p* value < 0.05). HC, higher compliance; LC, lower compliance; T0, baseline; T1, end; Total SCFA, sum of acetic acid, propionic acid, butyric acid, valeric acid, isobutyric acid, isovaleric acid and caproic acid; SCFA, short-chain fatty acid.

No differences were observed in the Shannon index or the observed species across the study (Figure 5A,B). The distribution of microbiota relative abundances is shown in Figure 5C,D) and Table S5. Those with LC presented an increase in Bacillota (51 to 61%), and the Oscillospiraceae family in this phylum increased from 2 to 3%. This was accompanied by a reduction in Prevotellaceae (Bacteroidota phylum) from 11 to 5%. Individuals with HC were characterized by a depletion of Actinomycetota (27 to 17%) and, within this phylum, of the families Bifidobacteriaceae, Coriobacteriaceae and Eggerthellaceae (12 to 7%, 9 to 5% and 5 to 4%, respectively) after the intervention. Regarding SCFA, only caproic acid was found to be 15% lower after the intervention in the LC group (Figure 6). 

### 3.5. Dietary Intake and Shifts of Microbiota Profile Composition among Individuals with Reduced Severity of Depressive Symptoms

**Figure 7:**
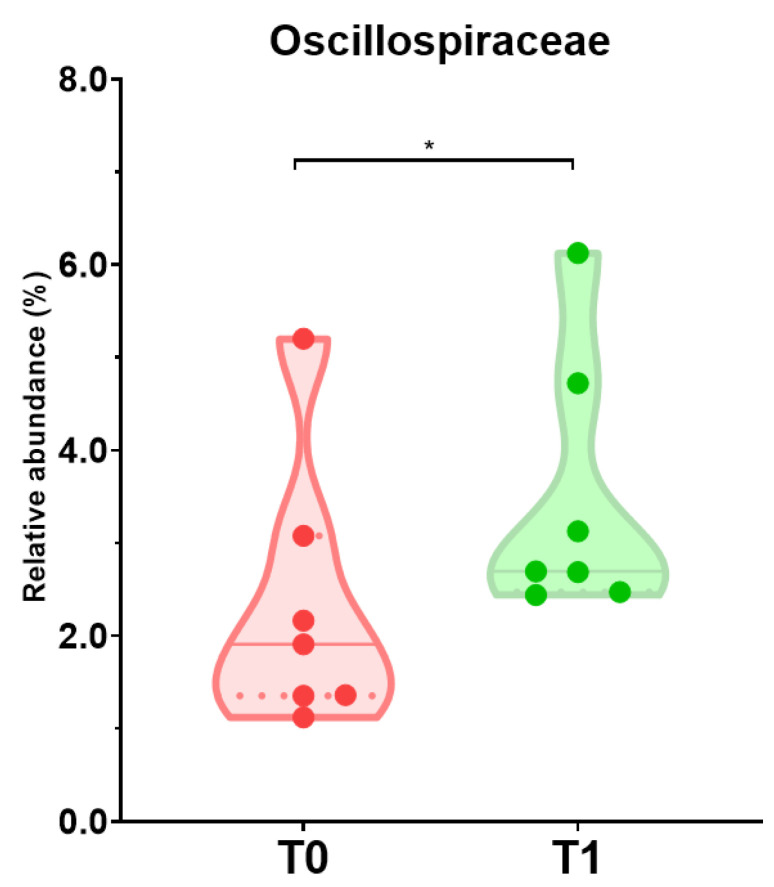
Relative abundance of Oscillospiraceae at baseline and after the intervention in individuals with lower compliance (LC) who presented successful amelioration of severity of depressive symptoms (n = 7). (*) Statistical differences were found by Wilcoxon test for comparisons within each group (T0 vs. T1) (*p* value < 0.05). T0, baseline; T1, end.

Both groups had improved BDI-II scores after the intervention (Figure 3). Subjects from the LC group with better BDI-II scores presented higher consumption of oils and fats (17 vs. 12 g/day), potatoes and tubers (96 vs. 25 g/day) (Table 5), together with a higher relative abundance of Oscillospiraceae (2.31 to 3.47%) (Figure 7, Table S6). In the case of the HC group, a reduced consumption of snacks (22 vs. 0 g/day) and soft drinks (317 vs. 0 g/day) was observed for those individuals with better BDI-II scores (*p* value 0.083) (Table 5), together with shifts in the microbiota profile. These modifications were in line with the general profile change observed in the whole HC group with the intervention (Figure 5C,D): reduced abundances of Actinomycetota and the families Coriobacteriaceae and Eggerthellaceae, and higher abundances of Ruminococcaceae (*p* value 0.043).

## 4. Discussion

The present work highlights the impact of a nutrition education intervention in a low-income group of adults on different health-related parameters and gut microbiota profiles. 

The starting point was marked by a dietary pattern with a low consumption of vegetables, fruits, and legumes in comparison with the recommendations (at least 400 g/day of vegetables and fruits and 50 g/day of legumes) [70,71]. Alcohol consumption was rarely present in the studied population (12% of the sample), in contrast to previous studies, analyzing the consumption of this toxic as related to the socio-economic status, although a similar proportion of regular smokers was found (24%) [9].

After the intervention, while 64% of the volunteers made between zero and two modifications in the PREDIMED items, 35% increased at least three criteria. This change was characterized by a greater intake of vegetables together with a reduction in the consumption of cereals and processed meats. In consequence, higher levels of some food-derived bioactive dietary components were achieved. Whereas the level of intake of starch was depleted, the ingestion of (poly)phenols, such as lignans, was tripled. High intakes of bioactive dietary components, such as fiber and (poly)phenols, are considered as possible protective factors against non-communicable diseases [17,34], this effect being partially linked to microbiota modulation [72]. In this regard, increased abundance of *Ruminococcus* (Bacillota phylum) and a reduced proportion of Actinomycetota, and the families Bifidobacteriaceae, Coriobacteriaceae and Eggerthellaceae, which belong to this phylum, were observed in the group of volunteers showing HC to the intervention. These results are in agreement with previous studies reporting a lower abundance of Actinomycetota with low-fat and high-fiber diets [73]. Also, fructo-oligosaccharides, fiber and inulin derived from flour-based products, such as cereals, have been shown to increase the relative abundance of *Bifidobacterium* [74]. Therefore, it is plausible that the reduction in the consumption of cereals and processed meat, together with the increase in lignans, could be associated with the shifts in this microbial genus. (Poly)phenols have been shown to inhibit the growth of this genus [75], and lower abundances have already been associated with a higher consumption of (poly)phenols and processed meats in previous works [76]. 

Depleted abundances of Coriobacteriaceae could be due to the reduced consumption of processed meats. Previous studies have also shown an association between this family, particularly the genus *Senegalimassilia* (also depleted), with starch dietary consumption, whereas starch and *Slackia* (Eggerthellaceae family) have also been associated in previous works [39]. Also, the increased levels of Ruminococcaceae, among other taxa with an affinity for polysaccharides, have been associated with an MD pattern [21]. The intervention did not significantly affect the alpha diversity of the microbiota, which is consistent with the results of a recent review on MD and microbiota [21]. 

To our knowledge, this is the first study analyzing the impact of a nutrition education intervention on xenobiotics consumption in a sample of low-income individuals. The intervention was effective in decreasing PAHs and nitrosamines. On the contrary, and probably resulting from the higher intake of vegetables during the intervention, an increased intake of nitrates was observed in the entire sample. These results are of great interest in order to strengthen healthy dietary interventions in this population group. According to our initial hypotheses, dietary intervention affects the BDI-II score and gut microbiota composition [77,78,79,80]. The depressive severity score was ameliorated in both the LC and HC groups. Although the proportion of subjects showing improvement in the HC group was higher, a significantly improved BDI-II score was only obtained in the LC group. Among these participants, a higher intake of oils and fats, potatoes and tubers and higher relative abundance of Oscillospiraceae were found. Although some authors have found an association between increased relative abundances of this microbial family and depression, the evidence in the literature is still inconsistent [81,82,83,84], and depression may remain constant after dietary interventions, according to previous research [85]. In all groups of individuals (LC and HC) an improvement in taste discrimination, both sweet and salty, was observed.

The LC group showed higher discrimination for sweet after the intervention without associated changes in microbial profile. Although it has been reported that sensitivity and discrimination tests can be affected by alcohol consumption, smoking, age, gender, or BMI [86,87,88,89,90,91,92], these variables remained constant across the intervention. 

For the interpretation of our data, it is important to keep in mind that this segment of the population is often unable to freely choose their dietary pattern. The financial budget provided to each volunteer along with the complex low socio-economic environment in which the people involved live, the limited sample size, and the short duration of the dietary intervention limit the strength and the potential impact of results obtained. 

## 5. Conclusions

In conclusion, this nutrition education intervention for materially deprived subjects demonstrated changes in dietary intake towards healthier food options and lowered the consumption of potentially carcinogenic compounds. Accompanied by shifts in fecal microbiota, this work has shown the potential of a nutrition education intervention to improve the sensitivity and the severity of depressive symptoms. Further research is required to confirm the obtained results in this study. This would allow for the design of future strategic policies that would contribute to the optimal nutrition of materially deprived families through healthy, plant-based affordable interventions. Furthermore, despite the small sample size, to our knowledge, this is the first study analyzing the whole picture of the impact of a nutrition education intervention for materially deprived subjects on diet, bioactive and xenobiotics consumption, fecal microbiota, sensitivity and discrimination of flavors and mood depression.

## Figures and Tables

**Table 1:** General characteristics of the study sample.

	Total Sample n = 17
Age (years)	41 (34–50)
Gender	
Female	14 (82)
Educational level	
Primary	4 (24)
Secondary	4 (24)
Technical	7 (41)
University	2 (12)
Family size(n)	
1–2	6 (35)
3–4	9 (53)
≥5	2 (12)
Lifestyle	
Sleep (hours/day)	6 (5–7)
Physical activity (walking min/day)	60 (21–90)
IPAQ classification	
Low/inactive	3 (18)
Moderate	8 (47)
Vigorous	6 (35)
Total METs	2010 (1315–2772)
Smoking status	
Current smoker	4 (24)
Former smoker	2 (12)
Never smoker	11 (65)
Occasional alcohol consumption ^(a)^	6 (35)
Chronic conditions	
Respiratory diseases	9 (53)

Data are expressed as median (P_25_–P_75_) and n (%) at baseline. IPAQ, International Physical Activity Questionnaire; METs, metabolic equivalents of task. ^(a)^ Frequency of consumption was lower than twice a week.

**Table 2:** Daily intake of energy and main food groups at baseline and after the intervention according to the degree of compliance with the dietary intervention.

	LC n = 11		HC n = 6	
	T0	T1	T0	T1
Energy (Kcal/day)	1471.98 (999.59–1641.27)	1437.07 (1197.17–1576.69)	1524.55 (1262.86–1849.45)	1376.52 (1123.48–1621.64)
Food groups intake (g/day)				
Cereals and cereals products	123.30 (58.33–148.07)	119.97 (94.44–146.20)	117.87 (113.29–174.66)	56.73 (41.20–92.75) * †
Whole grain cereals	0.00 (0.00–0.00)	10.00 (0.00–23.33)	0.00 (0.00–32.50)	4.33 (0.00–30.00)
Milk and dairy products	293.53 (148.23–451.66)	245.90 (179.33–530.68)	123.67 (31.67–187.50)	116.48 (84.17–204.73) †
Meat and meat products	110.00 (93.99–172.48)	94.83 (69.17–175.16)	156.58 (108.83–206.13)	102.98 (76.67–156.83)
White meat	53.58 (33.30–107.95)	55.33 (5.58–108.91)	53.58 (53.33–80.09)	79.02 (0.00–108.91)
Red meat	27.08 (5.90–63.33)	31.25 (0.00–50.00)	38.96 (5.90–110.16)	43.96 (0.00–47.92)
Proccesed meat	13.33 (0.00–31.33)	23.75 (13.33–40.00)	11.67 (6.67–23.33)	0.00 (0.00–0.00) * †
Eggs	53.33 (21.33–69.93)	42.67 (26.67–76.00)	58.30 (45.43–72.00)	34.28 (20.67–42.67)
Fish	21.67 (0.00–33.75)	59.67 (43.33–84.00)	27.97 (0.00–75.00)	61.25 (13.33–94.62)
Seafood	0.00 (0.00–29.00)	0.00 (0.00–0.00)	0.00 (0.00–0.00)	0.00 (0.00–26.66)
Oils and fats	14.09 (10.50–23.99)	15.53 (11.14–19.11)	21.00 (12.33–25.57)	24.01 (17.00–25.28)
Vegetables	67.71 (34.73–190.00)	98.80 (56.83–111.73)	144.48 (43.37–207.33)	251.35 (169.67–357.00) * †
Legumes	11.67 (0.00–33.33)	12.77 (0.00–46.67)	16.94 (7.77–150.00)	35.00 (0.00–200.00)
Potatoes and tubers	46.14 (8.33–88.83)	62.86 (33.33–123.00)	37.97 (23.00–59.00)	79.42 (24.60–101.00)
Fruits	96.17 (62.50–170.35)	139.35 (42.34–164.58)	151.00 (39.60–336.40)	262.08 (114.76–383.94)
Nuts and seeds	0.00 (0.00–0.00)	0.00 (0.00–0.00)	5.00 (0.00–16.67)	8.34 (0.00–16.67) †
Sugar and sweets	10.00 (0.00–20.80)	11.67 (4.17–18.67)	11.42 (7.00–64.59)	4.67 (0.00–14.00)
Snacks	0.00 (0.00–0.00)	0.00 (0.00–0.00)	0.00 (0.00–0.00)	0.00 (0.00–11.67) †
Sauces and condiments	18.50 (1.70–24.30)	13.83 (2.75–20.46)	21.37 (5.37–31.13)	10.14 (1.67–21.26)
Other foods	0.00 (0.00–125.00)	0.00 (0.00–75.00)	5.00 (0.00–83.33)	15.00 (0.00–40.00)
Non alcoholic beverages ^(a)^	268.33 (126.67–333.33)	250.00 (183.33–517.67)	345.84 (187.50–403.33)	225.00 (66.67–366.67)
Soft drinks ^(a)^	0.00 (0.00–0.00)	0.00 (0.00–83.33)	0.00 (0.00–166.67)	0.00 (0.00–83.33)
Alcoholic beverages ^(a)^	0.00 (0.00–0.00)	0.00 (0.00–33.33)	0.00 (0.00–0.00)	0.00 (0.00–0.00)

Data are expressed as median (P_25_–P_75_). Statistical differences were found by Wilcoxon (*) and Mann–Whitney U tests (†) for comparisons within each group (T0 vs. T1) and between groups (T0 vs. T0 and T1 vs. T1), respectively (*p* value < 0. 05). ^(a)^ (mL/day). LC, lower compliance; HC, higher compliance; T0, baseline; T1, end.

**Table 3:** Daily intake of bioactive compounds at baseline and after the intervention according to the degree of compliance with the dietary intervention.

Bioactive Compounds	LC n = 11		HC n = 6	
	T0	T1	T0	T1
Total dietary fiber	9.06 (6.77–14.45)	11.37 (9.01–15.73)	13.70 (12.44–15.06)	15.25 (12.37–19.66)
Soluble dietary fiber	1.34 (0.80–1.78)	1.25 (1.01–1.90)	2.00 (1.44–2.29)	1.51 (1.25–2.94)
Insoluble dietary fiber	5.04 (3.72–8.09)	5.85 (4.30–7.63)	9.25 (7.11–11.97) †	7.40 (5.64–12.78)
Starch	17.46 (9.07–35.87)	9.70 (7.44–18.85)	35.19 (10.07–49.84)	1.83 (0.11–14.04) *
Celulose	1.92 (1.66–3.31)	2.25 (1.70–3.30)	3.19 (2.31–3.80) †	2.93 (2.36–4.30)
Klason lignine	0.87 (0.43–1.18)	0.83 (0.73–1.21)	1.47 (1.27–2.04) †	1.15 (0.74–1.54)
Hemicellulose				
Soluble hemicellulose	1.05 (0.47–1.26)	0.85 (0.65–1.31)	1.27 (1.09–1.53) †	0.62 (0.60–1.60)
Insoluble hemicellulose	1.63 (1.18–2.71)	1.87 (1.47–2.40)	2.89 (2.51–3.63)	2.09 (1.88–4.32)
Pectin				
Soluble pectin	0.36 (0.26–0.47)	0.50 (0.30–0.57)	0.56 (0.32–0.65)	0.79 (0.45–1.23)
Insoluble pectin	0.61 (0.39–0.94)	0.74 (0.44–0.92)	1.05 (0.59–1.28)	1.26 (1.05–1.72)
Total (poly)phenols	346.16 (206.13–1208.90)	907.60 (552.78–1082.57)	869.88 (738.35–1211.61)	841.02 (795.02–913.25)
Flavonoids	14.70 (6.30–117.65)	61.82 (45.13–110.77)	105.13 (16.54–183.03)	57.23 (18.16–142.95)
Phenolic acids	186.24 (80.77–361.26)	271.21 (177.11–573.50)	589.79 (226.37–888.03)	324.55 (272.85–643.02)
Lignans	13.33 (4.32–27.69)	8.45 (4.79–16.00)	9.51 (8.73–14.92)	31.96 (18.06–51.23) * †
Other (poly)phenols	7.48 (2.12–16.29)	7.52 (6.05–11.87)	12.49 (9.79–22.45)	9.93 (6.85–36.28)
Stilbenes	0.00 (0.00–0.03)	0.01 (0.00–0.07)	0.01 (0.00–0.02)	0.00 (0.00–0.02)

Data are expressed as median (P_25_–P_75_). Statistical differences were found by Wilcoxon (*) and Mann–Whitney U tests (†) for comparisons within each group (T0 vs. T1) and between groups (T0 vs. T0 and T1 vs. T1), respectively (*p* value < 0.05). LC, lower compliance; HC, higher compliance; T0, baseline; T1, end.

**Table 4:** Daily intake of food-derived xenobiotic compounds at baseline and after the intervention according to the degree of compliance with the dietary intervention.

Xenobiotics	LC n = 11		HC n = 6	
	T0	T1	T0	T1
Heterocyclic amines (ng/day)				
IQ	0.00 (0.00–0.00)	0.00 (0.00–0.00)	0.00 (0.00–0.00)	0.00 (0.00–0.00)
MeIQ	0.00 (0.00–0.00)	0.00 (0.00–0.00)	0.00 (0.00–0.00)	0.00 (0.00–6.84)
MeIQx	47.91 (0.00–65.45)	81.47 (10.66–112.83)	36.73 (7.08–154.13)	54.63 (8.00–84.08)
DiMeIQx	2.33 (0.00–23.35)	33.20 (0.00–66.40)	1.18 (0.00–70.46)	25.00 (16.00–37.99)
PhlP	7.00 (0.00–298.97)	580.97 (10.94–1162.04)	66.50 (0.00–1743.00)	397.55 (4.00–603.31)
Polycyclic aromatic hydrocarbons (ng/day)				
B(a)P	36.30 (21.80–50.40)	46.10 (24.40–63.80)	52.20 (33.20–68.90)	36.40 (35.20–41.40)
DiB(a)A	3.20 (2.00–5.10)	4.30 (2.00–6.60)	4.30 (2.50–22.60)	2.40 (1.20–3.50) *
Total PAHs	616.10 (266.10–1190.70)	623.50 (368.60–1092.80)	1108.20 (660.70–1347.00)	372.80 (134.80–727.80) *
Nitrates. nitrites and nitroso compounds (ng/day)				
Nitrates (mg/day)	30.62 (17.77–48.18)	67.45 (24.00–100.83) *	100.77 (27.73–119.99)	153.26 (126.99–182.58) * †
Nitrites (mg/day)	0.63 (0.31–1.06)	1.24 (0.65–1.81)	0.65 (0.43–0.78)	0.36 (0.30–0.83) †
NDMA	34.70 (3.20–85.30)	86.70 (44.20–112.70)	24.90 (19.80–34.70)	0.00 (0.00–90.50)
NPIP	25.30 (0.00–45.50)	36.10 (24.00–63.30)	15.80 (8.00–25.30)	0.00 (0.00–0.00) * †
NPYR	45.30 (0.00–68.00)	64.60 (29.00–105.30)	28.30 (11.50–45.30)	0.00 (0.00–0.00) †
Comb	0.00 (0.00–0.00)	0.00 (0.00–4.70)	0.70 (0.00–1.90)	0.00 (0.00–0.00)
Acrylamide (µg/day)	8.73 (6.44–11.62)	12.24 (7.70–20.21)	8.44 (7.22–10.44)	2.83 (1.51-6.13) * †

Data are expressed as median (P_25_–P_75_). Statistical differences were found by Wilcoxon (*) and Mann–Whitney U tests (†) for comparisons within each group (T0 vs. T1) and between groups (T0 vs. T0 and T1 vs. T1), respectively (*p* value < 0.05). B(a)P, benzo (a) pyrene; Comb, Combined nitroso compounds; DiB(a)A, dibenzo (a) anthracene; DiMelQx, 2-amino-3,4,8 trimethylimidazo (4,5,f) quinoxaline; HC, higher compliance; IQ, 2-amino-3-methylimidazo (4,5,f) quinoline; LC, lower compliance; MelQ, 2-amino-3,4 dimethylimidazo (4,5,f) quinoline; MelQx, 2-amino-3,8 dimethylimidazo (4,5,f) quinoxaline; NDMA, N-nitrosodimethylamine; NPIP, n-nitrosopiperidine; NPYR, n-nitrosopyrrolidine; PAH, polycyclic aromatic hydrocarbons; PhlP, 2-amino-1-methyl-6-phenylimidazo (4,5,b) pyridine; T0, baseline; T1, end.

**Table 5:** Daily intake of energy and food groups at the end of the study according to the degree of compliance with the dietary intervention and the improvement of depressive symptoms.

	LC		HC	
	Improved BDI-II Score n = 7	No Improvement of BDI-II Score n = 4	Improved BDI-II Score n = 5	No Improvement of BDI-II Score n = 1
Energy (kcal/day)	1437.07 (1977.06–1250.26)	1317.42 (1467.85–1196.86)	1255.91 (1497.14–1123.48)	2101.11 (2101.11–2101.11)
Food groups intake (g/day)				
Oils and fats	17.00 (15.53–21.16)	11.46 (6.41–13.01) *	24.15 (17.00–25.28)	23.86 (23.86–23.86)
Olive oil	13.33 (11.00–17.00)	6.89 (4.06–9.83)	16.33 (11.00–17.48)	15.45 (15.45–15.45)
Cereals and cereals products	119.97 (94.44–147.27)	117.06 (67.89–139.27)	46.27 (41.20–67.19)	136.44 (136.44–136.44)
Whole grain cereals	10.00 (0.00–24.17)	12.27 (4.44–19.50)	0.00 (0.00–30.00)	8.67 (8.67–8.67)
Milk and dairy products	398.34 (215.00–545.84)	176.75 (137.84–355.01)	99.63 (84.17–133.33)	204.73 (204.73–204.73)
Meat and meat products	94.83 (69.17–175.16)	125.87 (60.53–194.29)	128.2 (77.76–156.83)	0.00 (0.00–0.00)
White meat	55.33 (5.58–69.17)	82.12 (26.79–127.42)	80.28 (77.76–108.91)	0.00 (0.00–0.00)
Red meat	31.25 (0.00–58.33)	33.33 (13.33–45.00)	47.92 (40.00–47.92)	0.00 (0.00–0.00)
Proccesed meat	33.33 (10.00–41.33)	21.88 (16.67–25.63)	0.00 (0.00–0.00)	0.00 (0.00–0.00)
Eggs	64.00 (32.00–84.67)	34.67 (13.34–46.84)	25.88 (20.67–42.67)	106.00 (106.00–106.00)
Fish	45.83 (3.00–107.33)	59.67 (53.62–71.84)	90.00 (32.50–94.62)	13.33 (13.33–13.33)
Seafood	0.00 (0.00–19.33)	0.00 (0.00–0.00)	0.00 (0.00–0.00)	26.66 (26.66–26.66)
Vegetables	90.11 (48.83–107.67)	105.27 (77.82–112.70)	193.00 (169.67–309.70)	372.27 (372.27–372.27)
Legumes	12.77 (0.00–53.00)	11.67 (0.00–35.00)	23.33 (0.00–46.67)	200.00 (200.00–200.00)
Potatoes and tubers	95.67 (47.83–150.67)	25.00 (16.52–64.19) *	66.67 (24.60–92.17)	241.00 (241.00–241.00)
Fruits	118.68 (42.34–164.58)	144.68 (69.68–168.9)	372.20 (114.76–383.94)	151.96 (151.96–151.96)
Nuts and seeds	0.00 (0.00–0.00)	0.00 (0.00–0.00)	16.67 (0.00–16.67)	0.00 (0.00–0.00)
Sugar and sweets	11.67 (4.17–18.67)	7.00 (0.00–19.50)	9.33 (0.00–14.00)	0.00 (0.00–0.00)
Snacks	0.00 (0.00–0.00)	0.00 (0.00–0.00)	0.00 (0.00–0.00)	21.67 (21.67–21.67)
Sauces and condiments	13.83 (0.67–17.33)	22.28 (6.96–56.22)	5.10 (1.67–15.17)	27.56 (27.56–27.56)
Other foods	0.00 (0.00–83.33)	0.00 (0.00–37.50)	30.00 (0.00–40.00)	0.00 (0.00–0.00)
Non alcoholic beverages (mL/day)	250.00 (183.33–517.67)	276.67 (135.00–462.50)	200.00 (66.67–250.00)	366.67 (366.67–366.67)
Soft drinks (mL/day)	0.00 (0.00–0.00)	41.67 (0.00–151.67)	0.00 (0.00–0.00)	316.67 (316.67–316.67)
Alcoholic beverages (mL/day)	0.00 (0.00–66.67)	2.00 (0.00–18.67)	0.00 (0.00–0.00)	0.00 (0.00–0.00)

Data are expressed as median (P_25_–P_75_). Statistical differences were found by Mann–Whitney U tests (*) for comparisons within each (*p* value < 0.05). LC, lower compliance; HC, higher compliance.

## Data Availability

Not applicable.

## Supplementary Materials

The following supporting information can be downloaded at: https://www.mdpi.com/article/10.3390/nu15163537/s1. Table S1: Anthropometric parameters at baseline and after the intervention according to the degree of compliance with the dietary intervention; Table S2: Biochemical parameters at baseline and after the intervention according to the degree of compliance with the dietary intervention; Table S3: Beck Depression Inventory-II (BDI-II) categories of depressive symptoms and total score at baseline and after the intervention according to the degree of compliance with the dietary intervention; Table S4: Sensitivity thresholds and discrimination scores for salt and sweet at baseline and after the intervention according to the degree of compliance with the dietary intervention; Table S5: Microbiota diversity indexes and relative abundance profile composition at baseline and after the intervention according to the degree of compliance with the dietary intervention.; Table S6: Microbiota diversity indexes and relative abundance profile composition at baseline and after the intervention in those individuals with LC showing amelioration of depressive symptoms after the intervention.
