# Supplementary material for: Impact on Fecal Microbiota and Health-Related Markers of an Intervention Focused on Improving Eating Behavior in People at Risk of Food Insecurity
Source: Nutrients. 2023 Aug 11;15(16):3537. doi: 10.3390/nu15163537 (PMC10459582; doi:10.3390/nu15163537)
Supplement: Supplementary file 1 [file nutrients-15-03537-s001.zip › nutrients-2489065-supplementary.pdf]

Supplementary Material

Table S1. Anthropometric parameters at baseline and after the intervention according to the degree of compliance with the dietary intervention

	LC n=11		HC n=6	
	T0	T1	T0	T1
Weight (Kg)	85.00 (56.60 - 92.60)	86.00 (56.80 - 94.40)	69.70 (59.20 - 85.40)	69.00 (58.40 – 86.00)
BMI (Kg/m^2)	30.18 (20.79 - 36.17)	30.11 (21.41 - 36.93)	25.58 (22.56 - 33.78)	25.65 (22.81 - 37.72)
Underweight (≤18.5)	1 (9)	0 (0)	0 (0)	0 (0)
Normal weight (18.5–24.9)	2 (18)	3 (27)	3 (50)	3 (50)
Overweight (25.0–29.9)	2 (18)	2 (18)	1 (17)	1 (17)
Obese (≥30.0)	6 (55)	6 (55)	2 (33)	2 (33)
Total body fat	9 (82)	9 (82)	6 (100)	6 (100)
(%)	46.00 (29.00 - 58.00)	45.00 (28.00 -57.00)	32.00 (27.00- 49.00)	34.50 (31.00 - 58.00)*
WHR	7 (64)	7 (64)	6 (100)	6 (100)
	0.85 (0.80 - 0.91)	0.81 (0.78 - 0.83)	0.86 (0.83 - 0.95)	0.88 (0.86 - 0.91)†

Data is expressed as median (P_{25} – P_{75}) and n (%). Statistical differences were found by Wilcoxon or Mc Nemar test (*) for continuous and categorical variables within each group (T0 *vs*. T1) and by Mann-Whitney U and Fisher test (†) for comparisons in-between (T0 *vs*. T0 and T1 *vs*. T1) (p value < 0.05). LC, lower compliance; HC, higher compliance; T0, baseline; T1, end; WHR, waist-hip ratio.

Table S2. Biochemical parameters at baseline and after the intervention according to the degree of compliance with the dietary intervention

(mg/dl)	LC n=11		HC n=6	
	T0	T1	T0	T1
Glucose	89.09 ± 5.82	92.60 ± 13.98	88.17 ± 13.63	90.00 ± 5.93
Total cholesterol	195.36 ± 28.54	188.70 ± 43.27	228.67 ± 44.97	229.83 ± 43.13
HDL	52.45 ± 16.49	53.50 ± 16.06	64.17 ± 14.16	63.00 ± 17.27
LDL	115.18 ± 21.38	108.20 ± 24.89	142.00 ± 38.30	141.33 ± 31.91
Triglycerides	138.91 ± 83.38	135.50 ± 94.81	112.33 ± 27.78	127.83 ± 45.74
Uric acid	4.55 ± 1.04	4.32 ± 0.91	4.37 ± 1.26	4.30 ± 1.06
Creatinine	0.70 ± 0.11	0.73 ± 0.16	0.64 ± 0.13	0.68 ± 0.16
Iron (μg/dl)	80.09 ± 39.36	66.90 ± 27.31	82.67 ± 24.81	89.67 ± 28.38

Data is expressed as mean ± sd. HC, higher compliance; HDL, high-density lipoprotein; LC, lower compliance; LDL, low-density lipoprotein; T0, baseline; T1, end.

Table S3. Beck Depression Inventory-II (BDI-II) categories of depressive symptoms and total score at baseline and after the intervention according to the degree of compliance with the dietary intervention

	LC n=11		HC n=6	
	T0	T1	T0	T1
**Categories**				
Minimal (≤ 13)	4 (36)	8 (73)	2 (33)	4 (67)
Mild (14 - 19)	5 (45)	2 (18)	2 (33)	0 (0)
Moderate (20 - 28)	1 (9)	0 (0)	0 (0)	1 (17)
Severe (≥ 29)	1 (9)	1 (9)	2 (33)	1 (17)
**Total score**	14 (11 - 18)	9 (4 - 14)*	18 (8 - 37)	11 (2 - 26)

Data is expressed as median (P_{25} – P_{75}) and n (%). (*) Statistical differences were found by Wilcoxon or Mc Nemar test (p value < 0.05). HC, higher compliance; LC, lower compliance; T0, baseline; T1, end.

Table S4. Sensitivity thresholds and discrimination scores for salt and sweet at baseline and after the intervention according to the degree of compliance with the dietary intervention.

	LC n=8		HC n=6	
	T0	T1	T0	T1
**Sensitivity (mM)**				
Salt	10.00 (7.50 - 15.00)	10.00 (5.00 - 12.50)	12.50 (10.00 - 50.00)	10.00 (10.00 - 10.00)
Sweet	15.00 (5.00 - 30.00)	15.00 (10.00 - 22.50)	15.00 (15.00 - 15.00)	15.00 (5.00 - 15.00)
**Discrimination (score)**				
Salt	3.00 (2.50 - 3.50)	3.50 (2.50 - 4.50)	2.00 (2.00 - 2.00)†	3.50 (3.00 - 4.00)
Sweet	3.00 (3.00 - 4.50)	4.00 (3.50 - 4.50)*	3.00 (2.00 - 4.00)	4.00 (2.00 - 5.00)

Data is expressed as median (P_{25} – P_{75}). Statistical differences were found by Wilcoxon (*) and Mann-Whitney U tests (†) for comparisons within each group (T0 *vs*. T1) and in-between (T0 *vs*. T0 and T1 *vs.* T1), respectively (p value < 0.05). HC, higher compliance; LC, lower compliance; T0, baseline; T1, end.

Table S5. Microbiota diversity indexes and relative abundance profile composition at baseline and after the intervention according to the degree of compliance with the dietary intervention.

	LC n=11		HC n=6	
	T0	T1	T0	T1
Diversity				
Observed species	711.64 ± 132.53	635.55 ± 142.26	800.33 ± 256.64	624.00 ± 93.88
Shannon index	5.80 ± 0.80	6.13 ± 0.35	6.14 ± 0.63	5.85 ± 0.34
Taxa (%)				
Bacillota	51.33 ± 11.45	60.86 ± 8.44*	48.38 ± 9.70	55.80 ± 9.02
Ruminococcaceae	11.70 ± 3.12	12.53 ± 3.07	11.12 ± 2.92	13.72 ± 2.13*
Oscillospiraceae	2.46 ± 1.31	3.42 ± 1.19*	2.69 ± 0.71	2.21 ± 0.73†
Clostridia_UCG-014	1.79 ± 1.61	0.71 ± 0.35*	0.60 ± 0.25†	0.64 ± 0.30
Actinomycetota	30.88 ± 14.75	23.93 ± 6.94	27.13 ± 12.51	16.56 ± 9.51*
Bifidobacteriaceae	17.08 ± 15.86	10.10 ± 6.46	12.16 ± 11.65	6.99 ± 7.57*
Bifidobacterium	17.08 ± 15.86	10.10 ± 6.46	12.16 ± 11.65	6.99 ± 7.57*
Coriobacteriaceae	8.93 ± 5.00	9.08 ± 4.87	8.88 ± 7.33	5.02 ± 3.19*
Senegalimassilia	1.60 ± 1.25	1.17 ± 0.39	1.88 ± 1.12	1.02 ± 0.77*
Collinsella	8.76 ± 5.00	8.97 ± 4.95	8.24 ± 7.54	4.38 ± 3.47*†
Eggerthellaceae	3.99 ± 2.10	3.66 ± 1.07	5.11 ± 2.13	3.55 ± 1.94*
Slackia	1.02 ± 0.71	1.03 ± 0.63	1.96 ± 1.28†	1.23 ± 0.99*
Bacteroidota	15.81 ± 9.41	13.05 ± 6.09	20.76 ± 4.10	25.01 ± 14.40
Prevotellaceae	10.90 ± 7.95	4.83 ± 2.71*	12.15 ± 4.73	19.67 ± 16.58
Prevotella	10.18 ± 7.64	4.25 ± 2.55*	11.24 ± 4.52	17.95 ± 15.24
UCG-002	1.28 ± 0.81	1.82 ± 0.91*	1.26 ± 0.39	1.20 ± 0.43

Data is expressed as mean ± sd. Statistical differences were found by Wilcoxon (*) and Mann-Whitney U tests (†) for comparisons within each group (T0 *vs*. T1) and in-between (T0 vs. T0 and T1 vs. T1), respectively (p value < 0.05). Only taxa with greater relative abundance than 1% in at least two samples showing significant changes after the intervention were considered in the analysis. LC, lower compliance; HC, higher compliance; T0, baseline; T1, end.

Table S6. Microbiota diversity indexes and relative abundance profile composition at baseline and after the intervention in those individuals with LC showing amelioration of depressive symptoms after the intervention.

	T0<br>n=7	T1<br>n=7
Diversity		
Observed species	5.69 ± 0.89	6.05 ± 0.40
Shannon index	694.71 ± 123.01	679.29 ± 169.79
Taxa (%)		
Bacillota	51.42 ± 11.23	58.90 ± 8.32
Lachnospiraceae	19.59 ± 5.37	21.48 ± 3.37
Agathobacter	5.71 ± 2.46	5.90 ± 1.96
Blautia	3.47 ± 1.39	4.02 ± 1.74
Dorea	1.30 ± 0.46	1.54 ± 0.28
Ruminococcus torques group	1.07 ± 0.57	1.21 ± 0.38
Veillonellaceae	3.41 ± 2.29	2.80 ± 2.05
Dialister	2.00 ± 1.50	1.96 ± 2.20
Megasphaera	1.34 ± 2.20	0.74 ± 1.01
Oscillospiraceae	2.31 ± 1.44	3.47 ± 1.42*
Faecalibacterium	4.73 ± 2.02	4.95 ± 2.31
Subdoligranulum	3.41 ± 0.46	3.89 ± 1.22
Peptostreptococcaceae	2.15 ± 1.08	1.85 ± 1.27
Romboutsia	1.69 ± 0.80	1.22 ± 0.78
Coprobacillaceae		
Catenibacterium	3.30 ± 2.85	4.80 ± 4.53
Streptococcaceae	2.12 ± 2.37	4.58 ± 3.54
Streptococcus	2.12 ± 2.38	4.57 ± 3.54
Clostridia_UCG-014	1.52 ± 1.03	0.81 ± 0.40
Eubacteriaceae		
Eubacterium coprostanoligenes group	1.27 ± 0.39	1.66 ± 0.56
Lachnospiraceae NK4A136 group	1.31 ± 0.96	1.10 ± 0.92
Erysipelotrichaceae	1.10 ± 0.67	1.76 ± 0.91
Holdemanella	0.86 ± 0.60	1.52 ± 0.91
Actinobacteriota	30.59 ± 17.21	26.25 ± 6.28
Coriobacteriaceae	9.18 ± 6.31	10.65 ± 5.36
Collinsella	8.98 ± 6.30	10.63 ± 5.35
Senegalimassilia	1.87 ± 1.53	1.08 ± 0.30
Bacteroidota	16.10 ± 11.40	12.83 ± 7.25
Bifidobacteriaceae	16.43 ± 17.76	11.04 ± 7.06
Bifidobacterium	16.42 ± 17.76	11.04 ± 7.06
Prevotellaceae	11.76 ± 9.70	4.20 ± 1.83
Prevotella	11.21 ± 9.39	3.68 ± 1.80
Ruminococcaceae	11.59 ± 2.61	11.91 ± 3.58
Eggerthellaceae	4.15 ± 2.55	3.38 ± 0.65
Erysipelatoclostridiaceae	3.69 ± 2.87	5.00 ± 4.51
Bacteroidaceae	3.08 ± 1.12	6.50 ± 6.07
Bacteroides	3.08 ± 1.12	6.50 ± 6.07

Data is expressed as mean ± sd. Statistical differences were found by Wilcoxon (*) for comparisons within each group (T0 *vs*. T1) (p value < 0.05). Only taxa with greater relative abundance than 1% in at least two samples were considered in the analysis. LC, lower compliance; HC, higher compliance; T0, baseline; T1, end.
